# The multiple pathways by which self-control predicts behavior

**DOI:** 10.3389/fpsyg.2013.00849

**Published:** 2013-11-12

**Authors:** Martin S. Hagger

**Affiliations:** Health Psychology and Behavioural Medicine Research Group, Faculty of Health Sciences, School of Psychology and Speech Pathology, Curtin UniversityPerth, WA, Australia

**Keywords:** self-regulation, willpower, strength model, reflective process, impulsive process, implicit motivation

Good self-control, that is, an individual's capacity to override impulses, urges, temptations, desires, and ingrained habits, is adaptive as it allows people to engage in sustained, effortful behavior to attain long-term outcomes, often at the expense of short-term gains and gratification. Research has shown that good self-control is associated with academic attainment, good health, cohesive relationships, and career progression. In contrast, poor self-control is related to chronic conditions like cardiovascular disease and obesity, alcohol problems, eating disorders, financial debt, and unplanned pregnancy. In a recent meta-analytic review, de Ridder and colleagues (2012) demonstrated a small-to-medium effect for trait measures of self-control on behavioral outcomes across multiple life domains. The review provides evidence that self-control is positively associated with adaptive, desirable outcomes and negatively associated with maladaptive, undesirable outcomes, and most strongly related to behaviors classified as “habitual” or “automatic.”

The review also lends support for the predictions of numerous theories of self-control in which self-control is conceptualized as a trait or dispositional capacity that affects behaviors across multiple domains. Findings are consistent with recent a model that conceptualizes self-control as a limited resource, which allows for good self-control but leads to self-regulatory failure once depleted (Baumeister et al., 2007; Hagger et al., 2009, 2010). According to the model, greater levels of trait self-control means more resources are available and better capacity for self-control (Baumeister et al., 2006). Drawing from de Ridder et al.'s findings and previous research and theory on self-control, I propose a comprehensive model that outlines the multiple pathways by which trait self-control affects behavior. In the model, I present a set of specific, testable hypotheses of trait self-control-action relations that will provide a basis for future theoretical development and empirical research investigating the mechanisms and processes involved. In the current analysis, consistent with De Ridder et al. (2012), I regard self-control as an individual difference that reflects capacity and availability of resources to engage in goal-directed behavior and overcome impulses and habitual responses.

I propose four pathways by which self-control affects behavior (see Figure 1). The first is a direct link between self-control and behavior (P1). This reflects the consistent association between dispositional self-control and action observed in numerous studies of self-control (De Ridder et al., 2012). Direct effects of dispositional variables in models of intentional behavior, such as the theory of planned behavior, have been frequently identified, including the effects of personality on behavior (Rhodes et al., 2002, 2004). The direct effects are independent of motivational processes or *intentions*, a focal construct of the theory and one that is proposed to mediate all distal influences, such as personality and individual differences, on action. It has been proposed that such direct effects unmediated by intention reflect the influence of implicit, spontaneous factors on behavior (Hagger et al., 2006).

**Figure 1:**
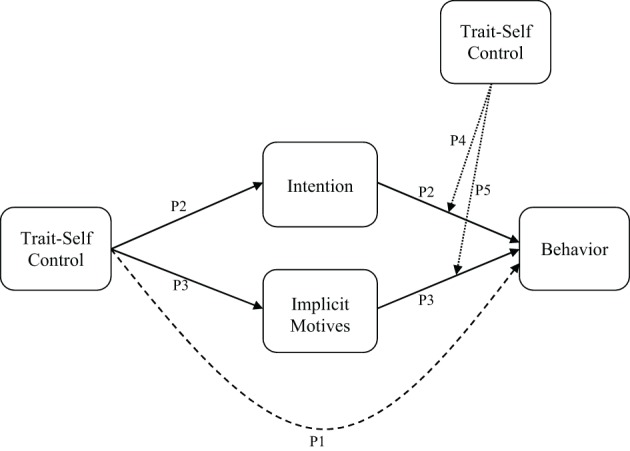
**Proposed pathways for trait self-control on behavior.** The direct broken path from self-control to behavior (P1) indicates a direct effect proposed to be mediated by the indirect effects through implicit motivation and intention/motivation. The broken lines from trait self-control to the intention-behavior (P4) and implicit motives-behavior pathways (P5) reflects moderation effects.

I propose two additional pathways by which dispositional self-control affects behavior. The first reflects more deliberative effects on action mediated by intention (P2). Tacit stored knowledge of capacity to engage in effortful action to attain a goal, and the ready availability of self-control resources, means individuals will be more likely to form plans and intentions to perform that action in future. Intentions, in turn, lead to subsequent goal-directed action. This pathway is akin to the “cold” or reflective system proposed in theories of self-control and action (e.g., Metcalfe and Mischel, 1999; Strack and Deutsch, 2004). The second pathway reflects the impulsive route by which self-control impacts action (P3). This pathway requires less deliberation and is likely driven by more spontaneous, automated responses. This is akin to the “hot” or impulsive route to action in which behavior is controlled by more spontaneous or automatized processes. It is also consistent with research adopting social-cognitive frameworks that have included implicit measures of motivation and demonstrated the effects of such measures on behavior independent of intention (Keatley et al., 2013). This pathway may reflect the extent to which an individuals' resource availability assists in determining the more impulsive, unconscious influences on action that have been well-rehearsed in the past and are therefore not dependent on intentional decision-making.

The proposed mediation effects (P2 and P3) reflect the extent to which self-control forms the basis of the deliberative and impulsive precursors of action. I also propose that self-control moderates the intention-behavior relationship (P4). This implies that the availability of self-control resources determines the extent to which individuals carry out their intentions. While self-control may be implicated in individuals' formation of intentions in the first place, their availability will dictate whether they have the propensity to execute them. This has been hypothesized by other investigators, indicating that individuals with good self-control will be more effective in engaging in intentional action because resource availability dictates the level of effort and investment that can be committed to pursuing the intended action (Hagger et al., 2009; Wills et al., 2011). Similarly, self-control resource availability will moderate the effect of implicit motives on action (P5). In this pathway, resources may determine the extent to which an individual is able to suppress impulsive determinants of action. Individuals with considerable resources will be more effective in suppressing this pathway. These interactive effects are proposed to be dynamic such that individuals with good self-control are more likely to enact their intentions and suppress their impulses, leading to a greater effect of P2 on behavior. Analogously, individuals with poor self-control are less likely to be able to engage in effortful planning and are less able to suppress the impulsive determinants of action, in which case the effect of P3 on behavior will be the most pervasive in the model.

The proposed model provides clear, testable hypotheses regarding the processes by which trait self-control influences behavior. Specifically, I have modeled the deliberative and spontaneous routes by which self-control may affect behavior through direct, indirect, and interactive pathways. I invite researchers to develop robust tests of these hypotheses using correlational and experimental methods to validate the model empirically (Hagger and Chatzisarantis, 2009). It is also important that these are tested in different behavioral contexts in which self-control is pertinent to success and failure.

## Author contributions

Martin Hagger conceived the ideas presented in the article and drafted the article.
